# Alcohol use disorder causes global changes in splicing in the human brain

**DOI:** 10.1038/s41398-020-01163-z

**Published:** 2021-01-05

**Authors:** Derek Van Booven, Mengying Li, J. Sunil Rao, Ilya O. Blokhin, R. Dayne Mayfield, Estelle Barbier, Markus Heilig, Claes Wahlestedt

**Affiliations:** 1 John P. Hussman Institute of Human Genomics, University of Miami Miller School of Medicine, Miami, FL 33136 USA; 2 Center for Therapeutic Innovation, University of Miami Miller School of Medicine, Miami, FL 33136 USA; 3 Department of Public Health Sciences, University of Miami Miller School of Medicine, Miami, FL 33136 USA; 4 Department of Psychiatry and Behavioral Sciences, University of Miami Miller School of Medicine, Miami, FL 33136 USA; 5 Jackson Memorial Hospital, Miami, FL 33136 USA; 6 Waggoner Center for Alcohol and Addiction Research, University of Texas at Austin, Austin, TX 78712 USA; 7 Center for Social and Affective Neuroscience, Department of Biomedical and Clinical Sciences, Linköping University, S-581 85 Linköping, Sweden

**Keywords:** Addiction, Molecular neuroscience, Epigenetics and behaviour

## Abstract

Alcohol use disorder (AUD) is a widespread disease leading to the deterioration of cognitive and other functions. Mechanisms by which alcohol affects the brain are not fully elucidated. Splicing constitutes a nuclear process of RNA maturation, which results in the formation of the transcriptome. We tested the hypothesis as to whether AUD impairs splicing in the superior frontal cortex (SFC), nucleus accumbens (NA), basolateral amygdala (BLA), and central nucleus of the amygdala (CNA). To evaluate splicing, bam files from STAR alignments were indexed with samtools for use by rMATS software. Computational analysis of affected pathways was performed using Gene Ontology Consortium, Gene Set Enrichment Analysis, and LncRNA Ontology databases. Surprisingly, AUD was associated with limited changes in the transcriptome: expression of 23 genes was altered in SFC, 14 in NA, 102 in BLA, and 57 in CNA. However, strikingly, mis-splicing in AUD was profound: 1421 mis-splicing events were detected in SFC, 394 in NA, 1317 in BLA, and 469 in CNA. To determine the mechanism of mis-splicing, we analyzed the elements of the spliceosome: small nuclear RNAs (snRNAs) and splicing factors. While snRNAs were not affected by alcohol, expression of splicing factor heat shock protein family A (Hsp70) member 6 (HSPA6) was drastically increased in SFC, BLA, and CNA. Also, AUD was accompanied by aberrant expression of long noncoding RNAs (lncRNAs) related to splicing. In summary, alcohol is associated with genome-wide changes in splicing in multiple human brain regions, likely due to dysregulation of splicing factor(s) and/or altered expression of splicing-related lncRNAs.

## Introduction

Alcohol use disorder (AUD) is a chronic condition characterized by a problematic pattern of alcohol use, which results in clinically significant impairment. In the United States, 14% of adults currently meet the criteria for AUD, 29% met AUD criteria once during their lifetime^1^; in addition, the prevalence of AUD is increasing^2^. Because of high prevalence and lack of efficient treatment modalities as well as due to association with multiple medical and psychiatric illnesses^3,4^, AUD causes a significant socioeconomic burden. The annual cost of AUD and alcohol-related disorders is ~$250 billion^5^. It has been recognized for a long time that damage to the brain inflicted by chronic alcohol use is severe and affects major domains of human life. In accordance to DSM-V, AUD is characterized by pervasive impairment in executive functions including lack of control over drinking, unsuccessful efforts to reduce alcohol intake, recurrent drinking in hazardous situations, etc.

Even though multiple brain regions might be involved in the pathogenesis of AUD, major sites are considered to be the frontal cortex, nucleus accumbens, and amygdala^6^. The frontal cortex is responsible for learning, decision-making, attention, and memory. Nucleus accumbens plays a critical role in processing rewarding stimuli, thus reinforcing pleasurable activities. Amygdala is a part of the limbic system which projects to nucleus accumbens and is mainly involved in the formation of emotional responses^7^. Alcohol crosses the brain and is capable of causing changes in gene expression in these regions, thus mediating the development of neurotoxicity, dependence, and tolerance. For example, 163 genes were altered in the superior frontal cortex in patients with AUD^8^. In the nucleus accumbens of rats given unlimited access to alcohol, 374 genes were altered, notably including many oncogenes^9^.

Post-transcriptional effects of alcohol are much less studied. Splicing is a nuclear post-transcriptional process of removing introns from pre-mRNA after which mature mRNA is produced and exported into the cytoplasm for translation (constitutive splicing). Alternative splicing is the process of selective incorporation of exons in mature mRNA transcripts which is responsible for transcriptomic and proteomic diversity. Perturbed splicing is implicated in a growing number of human diseases, including those related to the central nervous system. For example, cryptic splice site usage resulting in exon 7 skipping of PINK1 causes early-onset Parkinson’s disease, while increased inclusion of exon 10 in MAPT causes frontotemporal dementia with parkinsonism. Mechanistically, splicing is mediated by major and minor spliceosomes, nuclear machineries, each consisting of five small nuclear RNAs and dozens of splicing factors. Major spliceosome (also known as U2-dependent spliceosome) is composed of snRNAs U1 (snU1), snU2, snU4, snU5, and snU6 and is responsible for removal of ~99.5% introns. Minor spliceosome (also known as U12-dependent spliceosome) contains snU4atac, snU5, snU6atac, snU11, and snU12 and processes ~0.5% introns. Regulation of splicing and spliceosomes is poorly understood, but some evidence indicates that long noncoding RNAs (lncRNAs) might be implicated. LncRNAs are noncoding RNA molecules >200 nucleotides in length which are capable of interacting with both short RNAs and proteins and thus may serve as a “screwdriver” for spliceosome. It was shown that lncRNA Gomafu affects the formation of spliceosomes and inhibits splicing factor SF1^10^. Another lncRNA, MALAT1, interacts with serine/arginine splicing factors causing deregulation of splicing in a genome-wide fashion^11,12^. Data on splicing in AUD are very scanty. Alcohol intake was shown to be associated with a mis-splicing of specific genes such as AMPA receptors^13^ and GABA-B receptors^14^. Disruption of splicing on a somewhat broader scale was observed in the brain cortex of human fetuses exposed to alcohol^15^.

In this study, we set out to determine if alcohol affects splicing in the superior frontal cortex, nucleus accumbens, basolateral amygdala, and central nucleus of the amygdala. We found that mis-splicing in these regions occurs on a much broader scale than changes in gene expression, with thousands of transcripts being mis-spliced. Mechanistically, mis-splicing appears to be mediated by an increased expression of splicing factor heat shock protein family A (Hsp70) member 6 (HSPA6) and/or aberrant expression of lncRNAs related to splicing.

## Materials and methods

### Subjects

Postmortem human brain samples were obtained from the New South Wales Tissue Resource Centre at the University of Sydney and have been previously characterized^16^. Briefly, diagnosis of alcohol use disorder (AUD) was based on DSM-IV and was confirmed by physician interviews, review of hospital medical records, questionnaires to next-of-kin, and from pathology, radiology, and neuropsychology reports. Tissue samples were matched as closely as possible according to age, sex, postmortem interval, pH of tissue, disease classification, and cause of death. To be included as part of the alcohol-dependent cohort, subjects had to meet the following criteria: greater than 18 years of age, no head injury at the time of death, lack of developmental disorder, no recent cerebral stroke, no history of other psychiatric or neurological disorders, no history of intravenous drug use or polysubstance use, negative screen for human immunodeficiency virus and hepatitides B and C, and postmortem interval not exceeding 48 h.

Fresh-frozen samples of the superior frontal gyrus (SFC), nucleus accumbens (NA), basolateral amygdala (BLA), and central nucleus of the amygdala (CNA) were collected from each sample. All brain tissues were sectioned at 3-mm intervals in the coronal plane. There were no differences in the sectioning approach between the control and AUD groups.

### Sequencing of RNA from brain regions

RNA sequencing was performed as described recently^16^. The total RNA was extracted using mirVana™ miRNA Isolation Kit, with phenol (#AM1560, Thermo Fisher Scientific). RNA samples were DNAse-treated with DNA-free kit (#AM1906, Thermo Fisher Scientific), and ribosomal RNA was depleted using RiboMinus Eukaryote kit (Life Technologies). Two hundred and forty samples (30 alcoholics and 30 controls for each brain region) were processed using the TruSeq RNA Library Prep Kit v.2 and sequenced on the Illumina HiSeq 2 000 at the Genome Sequencing and Analysis Facility at The University of Texas at Austin. Paired-end libraries with an average insert size of 180 bp were obtained. Sequence read archives have submitted for all brain regions, and their accession numbers are as follows: PRJNA530758 (SFC), PRJNA551775 (NA), PRJNA551909 (BLA), and PRJNA551908 (CNA).

### Analysis of the transcriptome

Adapters were trimmed by TrimGalore. The alignment was performed with the STAR aligner (v.2.5.2a) against the hg19 human genome, and gene features were quantified using the GENCODE v.19 database. Raw counts were normalized into CPM values by edgeR in Bioconductor, and differential expression was calculated using a negative binomial model, with an FDR cutoff <0.05 used to define statistical significance.

### Analysis of mis-splicing events

Assessment of mis-splicing was performed as described previously^17^. The alignment was performed with the STAR aligner (v.2.5.2a) against the hg19 human genome. Resulting bam files from the STAR alignment were indexed with samtools for use by rMATS, as described elsewhere^18,19^. Briefly, rMATS pipeline used RNA sequencing reads which were mapped to different splice variants to estimate the isoform proportion, and a hierarchical framework was employed to simultaneously account for estimation uncertainty in individual replicates and variability among replicates. This software package has intrinsic features that focus on the interreplicate variability to identify underrepresented or overrepresented samples which are accomplished by counting the level of inclusion and exclusion of an event. FDR cutoff <0.05 was used to define statistical significance^20,21^.

### Rat model of alcohol use disorder based on the vapor chamber

Rats were placed in a vapor chamber with normal air for one week for habituation to a new environment (two rats per cage). Then alcohol vapor exposure was slowly increased in the course of 1 week until blood alcohol concentration (BAC) reached ~200 mg/dL. Rats were exposed to alcohol vapor for 7 weeks (5 days a week, 14 h/day) or left unexposed. BAC was measured once per week from one rat per cage (rats were alternating every other week). One week after the last alcohol exposure, animals were sacrificed, and brain samples were collected.

### Computational analysis of coding and noncoding features

Gene Set Enrichment Analysis database^22^ was used to obtain the list of spliceosomal proteins. Functional status of altered lncRNAs of interest was studied using “LncRNA Ontology” database^23^ which employs the pipeline based on transcriptional and epigenetic profiles of lncRNAs and protein-coding genes; default criteria set by developers were used to execute the ontology. Specifically, we mined for biological processes associated with specific lncRNA (individually), using the lncRNA-term associations at the confidence score of 100%. The resultant preliminary list included the query lncRNA, the inferred terms, the histone modification or expression used to infer the association, and other method(s) validating this association.

### Statistical analysis

The sample size in the current study was chosen based on our previous experience with similar studies, with a goal of ensuring adequate statistical power while including the least possible number of subjects. Statistical analysis between two groups was performed using two-tailed Student’s *t* test. Data were expressed as mean ± SEM unless otherwise stated. To compare the means between multiple groups, we employed two-way ANOVA which allows testing for interactions between two factors. Proportions in events in different groups were assessed using the chi-square test; rejection of the null hypothesis (that proportions are the same across groups) indicated that proportion is different in at least one group.

## Results

### Subjects

Demographics and pertinent clinical data are listed in Table 1. Patients with AUD and control subjects did not differ in age, gender, ethnic origin, or BMI; nor were there differences in brain weight and brain volume between the two groups (*P* > 0.05). Likewise, RNA integrity numbers of RNA samples were similar between control and AUD groups (*P* > 0.05, Supplementary Fig. 1).

**Table 1:** Baseline characteristics of patients with alcohol use disorder (AUD) and control subjects.

	Control, *n* = 30	AUD, *n* = 30
*Demographics*		
Age (SE)	57.5 (1.6)	57.5 (1.6)
Gender, males, *n* (%)	23 (77)	23 (77)
Ethnic origin, Europeans, *n* (%)	26 (87)	30 (100)
*Brain characteristics*		
Weight, g (SE)	1433 (25)	1381 (25)
volume, cm^3^ (SE)	1409 (24)	1364 (24)

*SE* standard error. Two-tailed Student’s *t* test was used to calculate the difference between patients with AUD and control subjects.

### Differential expression analysis

**Fig. 1:**
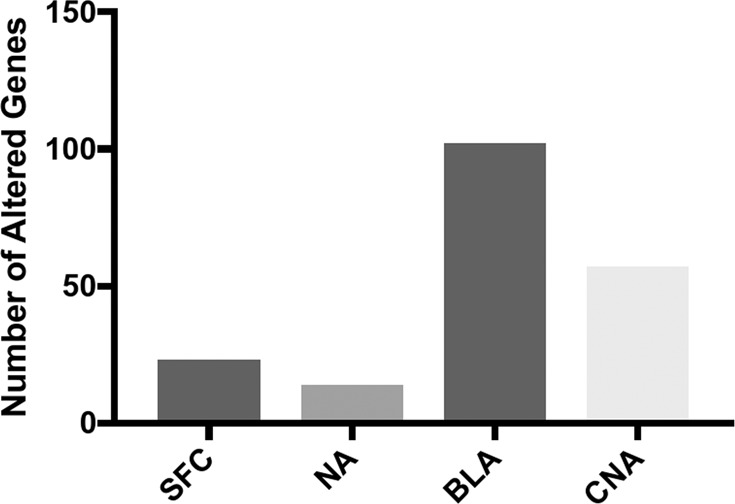
Differentially expressed genes in brain regions of patients with alcohol use disorder. SFC superior frontal cortex, NA nucleus accumbens, BLA basolateral amygdala, CNA central nucleus of the amygdala.

Analysis of expression of 57 820 total gene features (coding and noncoding) showed that AUD is associated with relatively moderate changes in brain transcriptome (with FDR cutoff <0.05). Thus, the most affected brain region was a basolateral amygdala (BLA), in which only 102 genes were differentially expressed. In the superior frontal cortex (SFC), nucleus accumbens (NA), and central nucleus of the amygdala (CNA), 23, 14, and 57 genes were altered, respectively (Fig. 1). The majority of affected genes (73.5%) were represented by protein-coding genes followed by long noncoding RNAs (20.9%) (Supplementary Fig. 2).

### Analysis of splicing in AUD

**Fig. 2:**
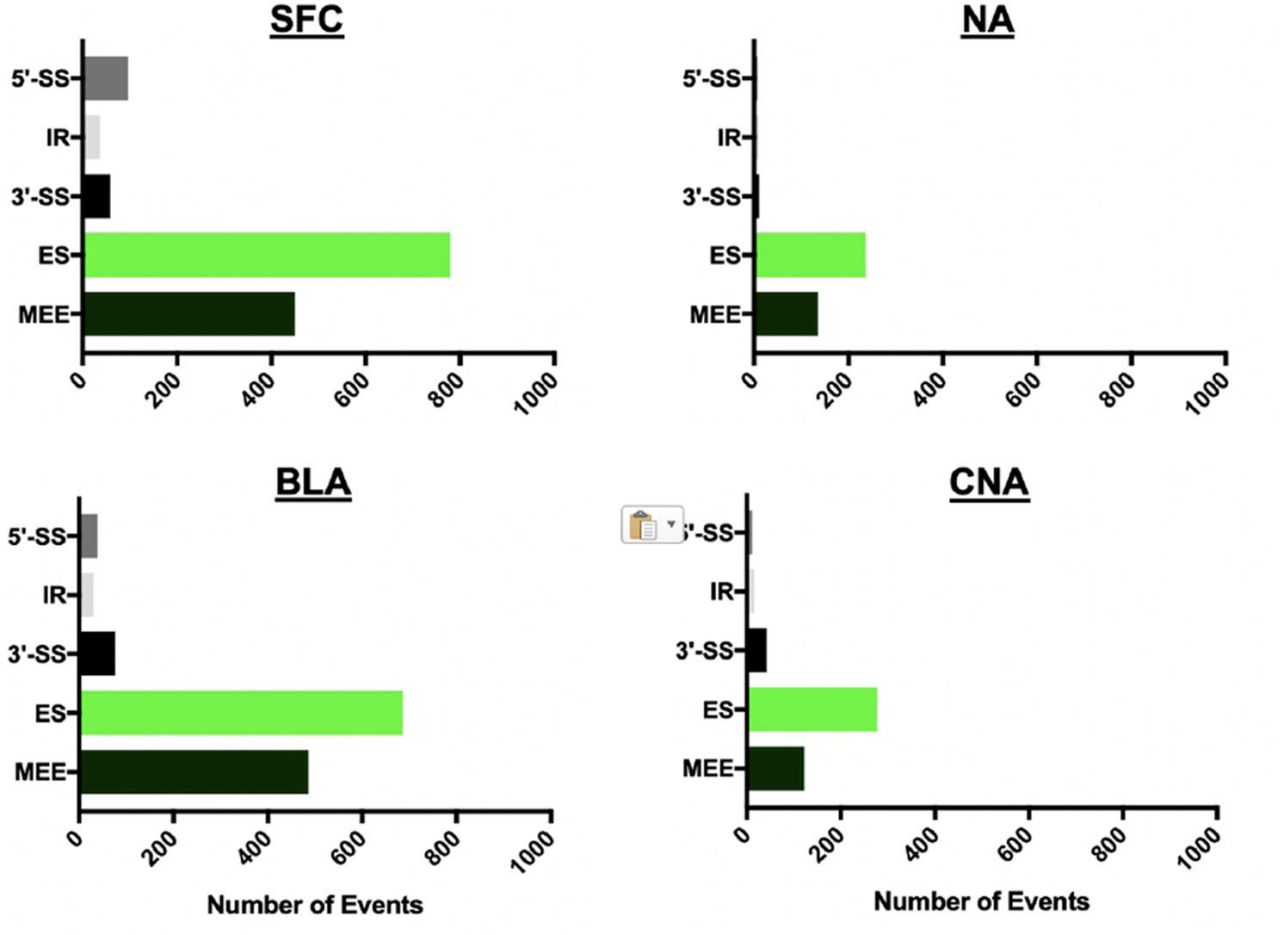
Mis-splicing patterns in brain regions of patients with alcohol use disorder. SFC superior frontal cortex, NA nucleus accumbens, BLA basolateral amygdala, CNA central nucleus of the amygdala; 5′-SS alternative 5′ splice site, IR intron retention, 3′-SS alternative 3′ splice site, ES exon skipping, MEE mutually exclusive exons.

The effect of alcohol on splicing in the brain has not been well studied. We employed MATS program^18^ which detects such mis-splicing events as 5′ alternative splice sites (5′-SS), 3′ alternative splice sites (3′-SS), intron retention (IR), exon skipping (ES), and mutually exclusive exons (MEE). In all four brain regions, these mis-splicing events were detected, with ES being the most common event and imbalance in MEEs—the second most common event (Fig. 2). Mis-splicing in AUD was much more profound than a differential expression: 1421 mis-splicing events were detected in SFC, 394 in NA, 1317 in BLA, and 469 in CNA. Frequency of skipped exons varied from 237 in NA to 779 in SFC. The frequency of imbalanced MEEs varied from 122 in CNA to 486 in BLA. The frequency of 5′-SS, 3′-SS, and IR was <100 in all four brain regions.

Of note, comparative analysis of mis-spliced genes did not show a significant overlap between brain regions, and only 14 genes were mis-spliced in all four regions (AKIP1, C11orf73, FLOT1, KIAA1841, NCALD, PDE4DIP, POMT1, PREPL, PR11-274B21.1, SORBS1, TPD52L1, VEZT, ZHX3, and ZNF638), suggesting a possibility of mis-splicing events occurring rather in a random manner.

### Expression of snRNAs in AUD

**Fig. 3:**
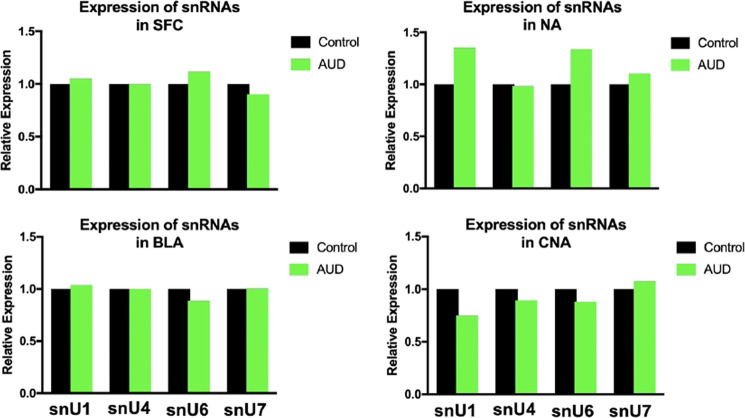
Expression of small nuclear RNAs (snRNAs) in the brain in alcohol use disorder (AUD). SFC superior frontal cortex, NA nucleus accumbens, BLA basolateral amygdala, CNA central nucleus of the amygdala. Two-tailed Student’s *t* test was used to compare the expression of snRNAs between two groups within each region. To compare the means between multiple groups, we employed two-way ANOVA which allows testing for interactions between two factors.

Next, we asked which mechanism may be responsible for such genome-wide alcohol-induced alterations in the splicing landscape. Splicing is governed mainly by major (U2-dependent) spliceosome which removes ~99.5% of all introns and consists of five snRNAs and multiple proteins, known as splicing factors. Furthermore, there is a minor (U12-dependent) spliceosome that is responsible for splicing of atypical snU12-type introns that constitute only ~0.5% of all introns in the human genome (process, also known as noncanonical splicing)^24^. We set out to determine if alcohol affects the expression of snRNAs and splicing factors in SFC, NA, BLA, and CNA. We started with small nuclear RNAs (snRNAs), as they are involved in the recognition of introns, formation of splicing complexes, and splicing reactions. Although data on AUD-induced changes in splicing have begun to accumulate^15^, the impact of alcohol on snRNA in the brain is not elucidated. We initially studied the expression of canonical snRNAs transcripts: snU1, snU4, snU6, and snU7 (expression of other canonical snRNA transcripts such as snU2, snU4atac, snU6atac, snU11, and snU12 was not reliably detected in our RNA sequencing output). We found that these 4 snRNAs were not affected in all studied brain regions (*P* > 0.05, Fig. 3). As each snRNAs has slightly divergent copies in the genome (sometimes referred to as snRNA pseudogenes), and some of them appear to be functional^25,26^, we additionally studied the expression of whole snRNAomes. As with canonical snRNAs, we found no difference in snRNAomes in all four regions between control subjects and patients with AUD (*P* > 0.05, Supplementary Fig. 3).

### Expression of splicing factors in AUD

**Fig. 4:**
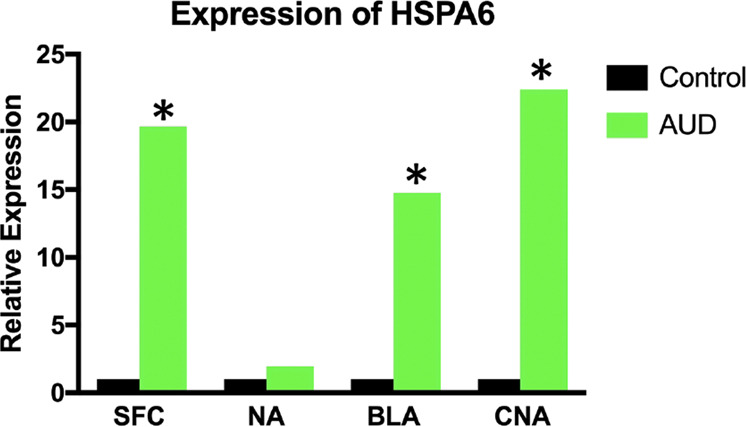
Expression of heat shock protein family A (Hsp70) member 6 (HSPA6) in the brain regions of patients with alcohol use disorder (AUD). SFC superior frontal cortex, NA nucleus accumbens, BLA basolateral amygdala, CNA central nucleus of the amygdala. Two-tailed Student’s *t* test was used to compare the expression of snRNAs between two groups within each region. *P < 0.05.

Next, we addressed if alcohol affects the expression of splicing factors. First, using Gene Set Enrichment Analysis resource, we have compiled the list of all spliceosomal factors. In total, 127 factors were included (Supplementary Table). Then, we undertook an animal study in which we treated rats with alcohol for 7 weeks (5 days a week, 14 h a day). At the end of the experiment, animals were sacrificed, RNA was isolated from the whole brain, and transcriptome was profiled using RNA sequencing. Among all splicing factors, only SF1 (splicing factor 1) was altered, demonstrating ~50% decrease in rats exposed to alcohol (data not shown). Of note, only four genes were differentially expressed in response to alcohol which differs much from relatively broader transcriptomic changes detected when brain regions were studied individually (Fig. 1). Such a discrepancy likely supports the concept that examination of the RNA profiles from the whole brain may be inaccurate due to region-specific transcriptomic alterations and that the RNA profiling in the brain should rather be done in a region-specific or/and lineage-specific manner. Next, we interrogated RNA sequencing datasets from the human study by aligning the list 127 spliceosomal genes with the list of genes differentially expressed in patients with AUD. Only one of the splicing factors, heat shock protein family A (Hsp70) member 6 (HSPA6), was detected in both lists, and its expression was consistently and markedly elevated in all brain regions. Thus, in SFC, HSPA6 mRNA levels were 19.7-fold increased. In NA, HSPA6 mRNA was approximately twofold upregulated, but this difference did not reach statistical significance. In BLA, HSPA6 mRNA concentrations were 14.8-fold increased. Finally, in the CNA, the greatest increase in HSPA6 mRNA was detected, 22.4-fold (*P* < 0.05, Fig. 4). Next, we checked if alcohol has a concerted effect on the spliceosomal proteome. Total expression of 127 spliceosomal genes was analyzed by averaging the expression of all individual genes, with mRNA levels of each gene in the control group assigned a value of 1, and expression of the same gene in the AUD group calculated relatively to a control group. We found no difference between control and AUD in all four regions (*P* > 0.05, Supplementary Fig. 4).

### Expression of lncRNAs in splicing in AUD

We observed that alcohol affected the expression of lncRNAs in all four brain regions (Table 2). Affected lncRNAs were represented by antisense lncRNAs, long intergenic noncoding RNAs (lincRNAs), and pseudogenes, at an approximately equal ratio. As lncRNA is known to be associated with splicing^10–12^, we set out to define the ontology of these affected lncRNAs. Major molecular functions were determined for each lncRNA, and integrative analysis revealed the functional relation of AUD-induced changes in lncRNAome to splicing and RNA processing (Table 3).

**Table 2:** Long noncoding RNAs altered in alcohol use disorder.

Type	Symbol	FC	FDR
*SFC*			
lincRNA	SNORD3C	37.6642755	3.62E-10
lincRNA	RP11-403B2.6	52.662752	0.007059185
lincRNA	RP11-13N12.1	0.1878297	0.026473897
lincRNA	SH3RF3-AS1	2.79438828	0.045879799
Pseudogene	HSPA7	4.15590188	0.026473897
*NA*			
lincRNA	RP11-13N12.1	0.05715863	0.005977715
lincRNA	RP11-300J18.1	0.06489013	0.026369459
Pseudogene	RP11-252O2.2	80.4983294	0.000136973
*BLA*			
Antisense	RP11-543H23.2	10.8243511	0.007934374
Antisense	RP11-258F1.1	2.37922452	0.015332852
Antisense	TBL1XR1-AS1	17.3312772	0.015532413
Antisense	AFAP1-AS1	3.18325329	0.017192359
Antisense	FAM201A	1.76319182	0.033190051
Antisense	RP11-61I13.3	2.0169569	0.034644143
Antisense	AC137932.6	4.48970088	0.046685483
lincRNA	SNORD3C	29.1509868	2.75E-09
lincRNA	RP11-13N12.1	0.05915464	0.001230207
lincRNA	RP11-638F5.1	5.0143603	0.001568731
lincRNA	FAM225B	2.90151166	0.006760463
lincRNA	LINC00313	3.27085457	0.011320488
lincRNA	RP4-723E3.1	4.54423735	0.036184405
lincRNA	RP11-1L9.1	0.53434789	0.0436353
lincRNA	RP11-713P17.3	1.92849923	0.046685483
Pseudogene	HSPA7	7.72353205	0.000344011
Pseudogene	RPLP0P2	3.4401232	0.003849772
Pseudogene	RP5-1033K19.2	17.9403402	0.00962731
Pseudogene	KRT16P2	10.884735	0.014255406
Pseudogene	CTD-2575K13.6	3.95176513	0.022758622
Pseudogene	AC000367.1	2.0252892	0.031275117
Pseudogene	RP11-299H22.5	7.12002207	0.039950948
*CNA*			
Antisense	RP11-543H23.2	15.2693924	0.006706822
Antisense	RP11-258F1.1	2.77641286	0.032959908
Antisense	RP5-1185I7.1	16.5547413	0.03784948
Antisense	RP11-350G8.5	3.19314475	0.039819746
Antisense	AC004066.3	0.45310563	0.040012906
Antisense	RP13-126C7.1	6.53582789	0.040012906
lincRNA	SNORD3C	16.6381473	2.43E-07
lincRNA	RP11-13N12.1	0.10266091	0.009545421
Pseudogene	HSPA7	13.9240757	1.48E-05
Pseudogene	RPLP0P2	3.56669803	0.029432652
Pseudogene	MTND6P6	2.05977967	0.047701809

*SFC* superior frontal cortex, *NA* nucleus accumbens, *BLA* basolateral amygdala, *CNA* central nucleus of the amygdala, *FC* fold change, *FDR* false discovery rate, *LincRNA* long intergenic noncoding RNA.

**Table 3:** Functional ontology of long noncoding RNAs (lncRNAs) altered in alcohol use disorder.

Biological process	Number of lncRNAs
mRNA splicing, via spliceosome	11
RNA processing	10
mRNA processing	10
Nuclear-transcribed mRNA poly(A) tail shortening	8
Nuclear-transcribed mRNA catabolic process	7

## Discussion

The salient findings of the current study are: (1) AUD causes genome-wide changes in splicing in the brain; (2) AUD markedly increases the expression of splicing factor HSPA6; (3) AUD affects lncRNAs which are functionally related to splicing.

In the past few years, there have been several studies on alcohol-induced changes in gene expression in the brain. For example, gene expression profiling on the prefrontal cortex identified 129 altered genes in patients with AUD^16^. Likewise, microarray analysis detected 163 differentially expressed genes in the superior frontal cortex in AUD^8^. However, to what extent alcohol can affect transcriptome posttranscriptionally via splicing remains unknown. In this study, we combined a conventional analysis of differential expression with the genome-wide assessment of mis-splicing events. Results of differential expression analysis were relatively comparable with previous studies. We found that alcohol affects gene expression on a moderate scale. Thus, the least affected region was the nucleus accumbens in which only 14 genes were affected, while the most affected region was the basolateral amygdala (102 genes). It is not known yet how alcohol affects gene expression in the brain. Besides splicing, which was the focus of this study, there are other molecular mechanisms that might be involved. For example, alcohol is associated with aberrant patterns of DNA methylation of CpG islands^27^ and changes in histone code^28^; alcohol is also capable of changing the expression of microRNAs which fine-tune the transcriptome in the cytoplasm^29^. Of note, as the extent of transcriptomic damage in our study was different across brain regions, it is possible that the mechanism of alcohol-induced changes may be region-specific and should likely be studied separately in each anatomical site.

Splicing takes a central place in cellular biology: constitutive splicing is responsible for the removal of introns, and alternative splicing generates proteomic diversity. We observed that while changes in gene expression were relatively modest across brain regions, mis-splicing events were observed on a much broader scale. In total, 3601 mis-splicing events have been detected, mostly skipping of exons and imbalance between mutually exclusive exons. To date, there were very few studies on the effect of alcohol on splicing, almost all of which focused on mis-splicing of specific genes. For example, alcohol was shown to be associated with a mis-splicing of AMPA receptors^13^ and GABA-B receptors^14^. Ethanol also affected alternative splicing of DRD2 (dopamine D2 receptor) in the pituitary^30^. Only one study highlighted relatively broad alcohol-induced changes in splicing. Kawasawa et al.^15^ found 382 alternative splicing events in the brain cortex of human fetuses exposed to alcohol. Qualitatively, eight mis-splicing events were detected: 5’ and 3’ alternative splice sites, mutually exclusive exons, intron retention, cassette exon, coordinate cassette exon, alternative first exon, and alternative last exon (exon skipping was likely not assessed by employed computational pipeline), with intron retention being the most frequent event. Differences between this and our study may possibly be attributed to different developmental stages of the brain (fetus vs adult) as well as to different bioinformatics approaches.

We found that AUD leads to a marked increase of splicing factor HSPA6 mRNA levels in the superior frontal cortex, basolateral amygdala, and central nucleus of the amygdala. HSPA6 is an inducible member of the family of heat shock proteins. HSPA6 is much less studied compared to other heat shock proteins, likely due to its relatively recent evolutionary emergence, as HSPA6 is present in the human genome but absent in mice and rats. As a result, no animal studies on HSPA6 are available. Studies on human neurons showed that in response to thermal stress HSPA6 targets nuclear speckles^31,32^. Nuclear speckles are structures enriched in splicing factors and viewed as compartments supplying splicing factors to transcription sites^33^. When heat shock occurs, nuclear speckles enlarge due to accumulation (“trapping”) of splicing factors^34^, which is part of transcriptional reprogramming of the stressed cell. One could hypothesize, therefore, that alcohol-induced increase in HSPA6 expression may represent stress-associated cellular response disrupting the normal functioning of nuclear speckles and consequently halting splicing. Of note, HSPA6 is mostly known for its involvement in carcinogenesis. Specifically, increased expression of HSPA6 was shown to be associated with a poorer prognosis in hepatocellular carcinoma^35^, an established alcohol-related cancer^3^. Even though alcohol is not generally considered to be associated with brain tumors, the trend to a higher incidence of brain tumors in heavy drinkers was detected in meta-analysis^36^. Since alcohol is associated with some cancers (hepatocellular carcinoma, head and neck squamous cell carcinoma, etc.) and splicing is clearly involved in carcinogenesis^37^, this study may provide novel mechanistic insight and suggest new directions in studying the possible link between AUD and brain tumors.

Another, post-transcriptional, mechanism of regulation of splicing may be represented by lncRNAs. Spliceosome represents gigantic machinery consisting of snRNAs and splicing factors and functionally coupled with gene expression^38^. Since lncRNAs can interact with DNA, RNA, and proteins, it is possible that they serve as a master regulator of the spliceosome. We interrogated lncRNAs and found that lncRNAs affected in AUD were functionally related to splicing. Recent evidence indicates that even individual lncRNA may cause marked changes in splicing. Thus, lncRNA MALAT1 which is upregulated in the brain of alcoholics^39^ interacted with splicing factors HNRNPF and HNRNPF1, changed levels of serine–arginine-rich splicing factors, and affected alternative splicing of hundreds of transcripts^40^. Alternative splicing was documented to be regulated by sno-lncRNAs, lncRNA flanked by small nucleolar RNA (snoRNAs)^41^ which are a family of conserved nuclear RNAs located in Cajal bodies or nucleoli and participating in snRNAs modifications. Several lncRNAs are able to interact with specific splicing factors^42^. A new exciting field is represented by circular RNAs (circRNAs), covalently closed single-stranded RNA complexes arising from backsplicing. These complexes are highly stable, and it is speculated that they might be capable of competing with pre-mRNA for spliceosome^43^.

In sum, we showed that AUD markedly perturbs splicing in the superior frontal cortex, nucleus accumbens, basolateral amygdala, and central nucleus of the amygdala in a genome-wide fashion. Mechanistically, mis-splicing may be mediated by increased expression of HSPA6 and/or altered expression of splicing-related lncRNAs.

## Supplementary information

Suppl figures

Suppl Table
