# Supplementary material for: Alcohol use disorder causes global changes in splicing in the human brain
Source: Transl Psychiatry. 2021 Jan 5;11:2. doi: 10.1038/s41398-020-01163-z (PMC7790816; doi:10.1038/s41398-020-01163-z)
Supplement: Supplementary file 1 — Suppl figures [file 41398_2020_1163_MOESM1_ESM.pdf]

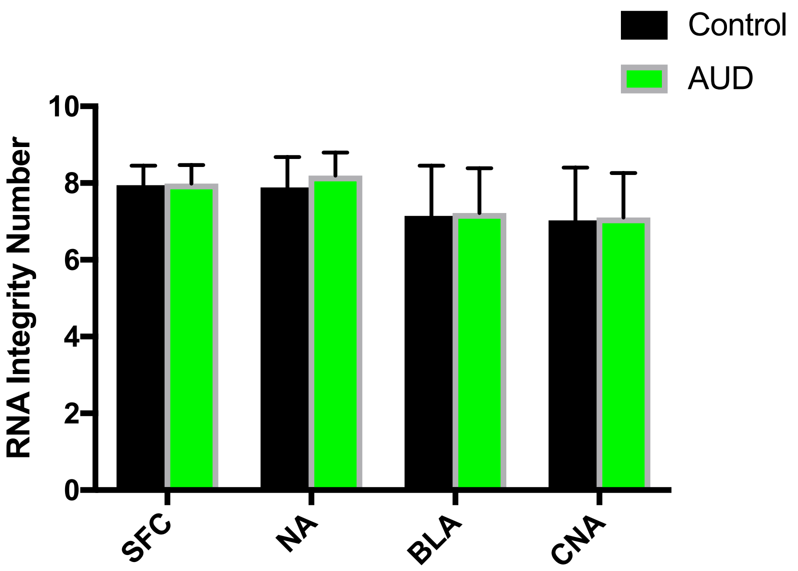

Supplementary Figure 1. RNA integrity numbers of RNA isolated from superior frontal cortex (SFC), nucleus accumbens (NA), basolateral amygdala (BLA), and central nucleus of amygdala (CNA) from control subjects and patients with alcohol use disorder (AUD). Data are presented as means with standard errors. $P > 0.05$, calculated by t test within each region.

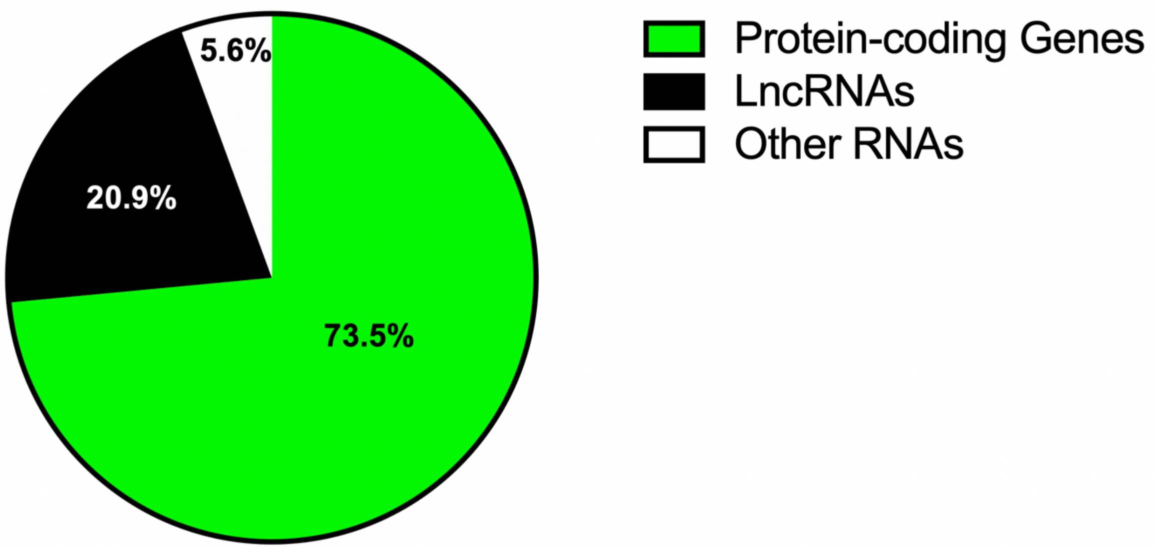

Supplementary Figure 2. Distribution of altered transcripts in the brain of patients with alcohol use disorder. LncRNAs – long non-coding RNAs.

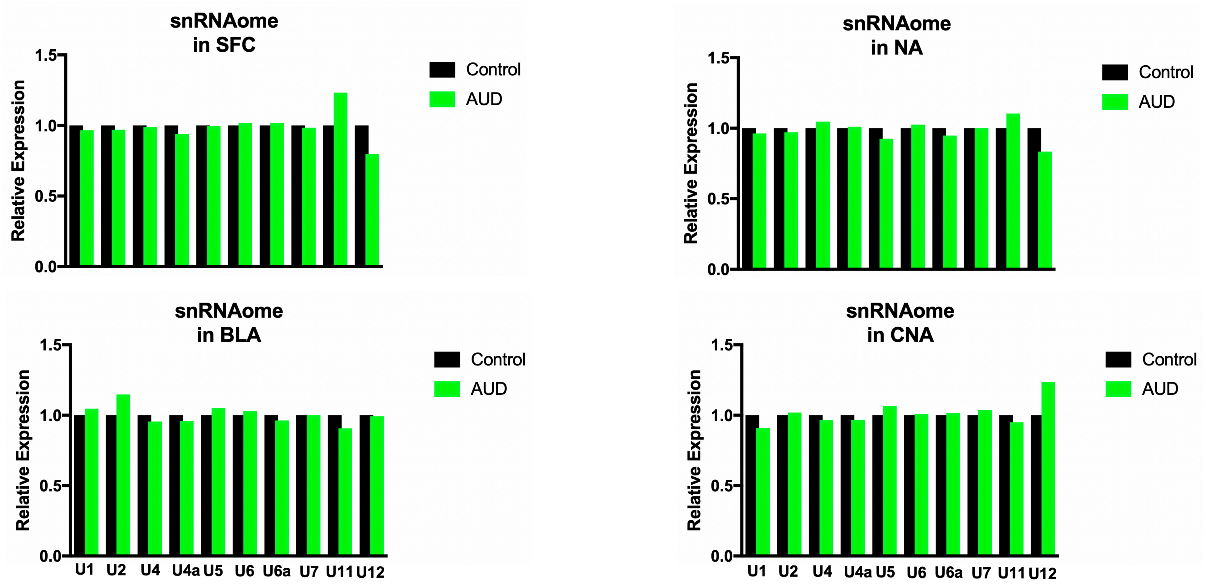

Supplementary Figure 3. SnRNAomes in the brain in of patients with alcohol use disorder (AUD). SFC – superior frontal cortex, NA – nucleus accumbens, BLA – basolateral amygdala, CNA – central nucleus of amygdala. Two-tailed Student's *t* test was used to compare the expression of snRNAs between two groups within each region. To compare the means between multiple groups, we employed two-way ANOVA which allows to test for interactions between two factors.

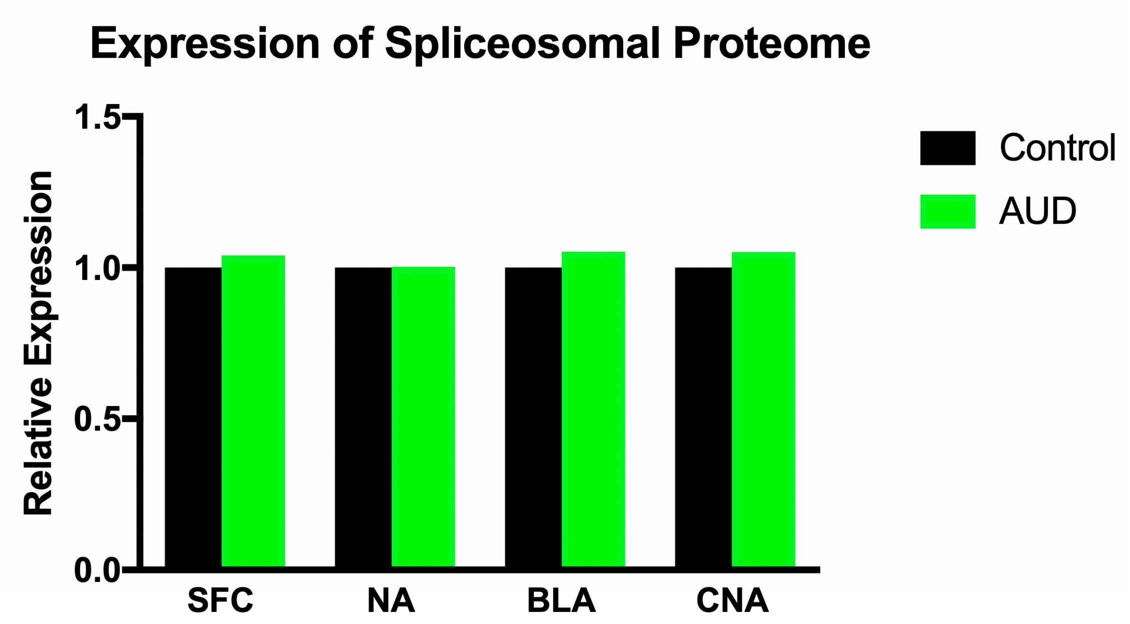

Supplementary Figure 4. Expression of spliceosomal proteome in the brain of patients with alcohol use disorder (AUD). SFC – superior frontal cortex, NA – nucleus accumbens, BLA – basolateral amygdala, CNA – central nucleus of amygdala. Two-tailed Student's *t* test was used to compare the expression of snRNAs between two groups within each region. To compare the means between multiple groups, we employed two-way ANOVA which allows to test for interactions between two factors.
