# Supplementary material for: Alcohol use disorder causes global changes in splicing in the human brain
Source: Transl Psychiatry. 2021 Jan 5;11:2. doi: 10.1038/s41398-020-01163-z (PMC7790816; doi:10.1038/s41398-020-01163-z)
Supplement: Supplementary file 2 — Suppl Table [file 41398_2020_1163_MOESM2_ESM.pdf]

Supplementary Table

List of Splicing Factors

ACIN1	LSM2	SF3A2	THOC1
AQR	LSM3	SF3A3	THOC2
BCAS2	LSM4	SF3B1	THOC3
BUD31	LSM5	SF3B14	THOC4
CCDC12	LSM6	SF3B2	TRA2A
CDC40	LSM7	SF3B3	TRA2B
CDC5L	MAGOH	SF3B4	TXNL4A
CHERP	MAGOHB	SF3B5	U2AF1
CRNKL1	NAA38	SLU7	U2AF2
CTNNBL1	NCBP1	SMNDC1	U2SURP
CWC15	NCBP2	SNRNP200	USP39
DDX23	NHP2L1	SNRNP27	WBP11
DDX39B	PCBP1	SNRNP40	XAB2
DDX42	PHF5A	SNRNP70	
DDX46	PLRG1	SNRPA	
DDX5	PPIE	SNRPA1	
DHX15	PPIH	SNRPB	
DHX16	PPIL1	SNRPB2	
DHX38	PQBP1	SNRPC	
DHX8	PRPF18	SNRPD1	
EFTUD2	PRPF19	SNRPD2	
EIF4A3	PRPF3	SNRPD3	
HNRNPA1	PRPF31	SNRPE	
HNRNPA1L2	PRPF38A	SNRPF	
HNRNPA3	PRPF38B	SNRPG	
HNRNPC	PRPF4	SNW1	
HNRNPK	PRPF40A	SRSF1	
HNRNPM	PRPF40B	SRSF10	
HNRNPU	PRPF6	SRSF2	
HSPA1A	PRPF8	SRSF3	
HSPA1B	PUF60	SRSF4	
HSPA1L	RBM17	SRSF5	
HSPA2	RBM22	SRSF6	
HSPA6	RBM25	SRSF7	
HSPA8	RBM8A	SRSF8	
ISY1	RBMX	SRSF9	
LOC120364	SART1	SYF2	
LOC653884	SF3A1	TCERG1	
